# Sprachentwicklungstest für zweijährige Kinder (2;0–2;11 Jahre) – Auswertung multizentrischer Daten von Kindern mit Cochleaimplantat nach bilateraler Versorgung: Eine retrospektive Längsschnittuntersuchung

**DOI:** 10.1007/s00106-024-01536-6

**Published:** 2024-12-10

**Authors:** Stefanie Kröger, Antje Aschendorff, Cynthia Glaubitz, Kerstin Kreibohm-Strauß, Dominique Kronesser, Yvonne Seebens, Barbara Streicher, Fabian Overlach, Stephanie Rother, Rainer Beck

**Affiliations:** 1 Klinik und Poliklinik für Hals-Nasen-Ohrenheilkunde, Sektion Implant Centrum Freiburg (ICF), Universitätsklinikum Freiburg, Medizinische Fakultät, Albert-Ludwigs-Universität Freiburg, Elsässerstraße 2n, 79110 Freiburg, Deutschland; 2 Hals-Nasen-Ohren-Klinik, Kopf- und Halschirurgie, CICERO Cochlear-Implant-Centrum, Uniklinikum Erlangen, FAU Erlangen-Nürnberg, Erlangen, Deutschland; 3 Cochlear Implant Centrum Wilhelm Hirte, Hannover, Deutschland; 4 Klinik und Poliklinik für Hals-Nasen-Ohrenheilkunde, Sächsisches Cochlear Implant Centrum (SCIC), Universitätsklinikum Carl Gustav Carus Dresden, Dresden, Deutschland; 5 Cochlear Implant Center (CIC) Rhein-Main der HSF gGmbH, Friedberg, Deutschland; 6 Klinik und Poliklinik für Hals‑, Nasen- und Ohrenheilkunde, Universitätsklinikum Köln, Cochlear Implant Centrum (CIK), Köln, Deutschland

**Keywords:** Verlaufsbegleitende Diagnostik, Spracherwerbsstörungen, Kongenitale Taubheit, Rehabilitation, Cochleaimplantat, Accompanying diagnostics, Speech development disorders, Congenital deafness, Rehabilitation, Cochlear implant

## Abstract

**Hintergrund:**

Ein zeitgerechter Spracherwerb ist eines der Kernziele der Rehabilitation bei Kindern, die mit einem Cochleaimplantat (CI) versorgt wurden. Verschiedene Testverfahren werden zur Beurteilung herangezogen, so auch der Sprachentwicklungstest für zweijährige Kinder (SETK-2). Alle Verfahren wurden an normalhörenden Kindern normiert, zusätzlich erfolgt die Auswertung teils nach Lebensalter, teils gemäß Zeitraum nach der CI-Versorgung (Höralter). In der vorliegenden Untersuchung wird momentan bestehende Praxis beschrieben und soweit möglich bewertet.

**Material/Methode:**

In einer multizentrischen retrospektiven Studie wurden von 5 CI-Centren 375 Datensätze des SETK‑2 von kongenital tauben und bilateral mit CI versorgten Kindern erhoben. Alle wurden vor Vollendung des 4. Lebensjahres versorgt, der Abstand betrug bei bilateraler CI-Versorgung weniger als 12 Monate.

**Ergebnisse:**

Alle Subtests der betrachteten Gruppen schnitten mit Ausnahme des Verstehens für Wörter in der Auswertung nach Hör- und Lebensalter signifikant schlechter als die Normstichprobe ab. Einzelergebnisse zeigten gleich gute oder sogar bessere Leitungen im Vergleich zur Normstichprobe. Je komplexer die geprüfte Leistung war, desto höher war der Anteil der auffälligen Testergebnisse. Der Zeitpunkt der Implantation zeigte keinen wesentlichen Einfluss auf die Testergebnisse.

**Schlussfolgerung:**

Die Auswertung des SETK‑2 sollte nach Lebensalter erfolgen, da sonst die Entwicklung im frühen Spracherwerb falsch eingeschätzt und Interventionen zu spät initiiert werden könnten. Außerdem lässt die Beurteilung nach Höralter die Kognition des Kindes außer Acht.

## Abstract (en)

**Background:**

Age-appropriate speech development is a primary aim of the rehabilitation of children treated with cochlear implants (CI). Various assessment tools are available, including the speech development test for 2‑year-olds (SETK-2). All tests are normalized to normal-hearing children; additionally, results are evaluated according to age and the duration of CI (hearing age). The present study presents current practices and evaluates them as far as is possible.

**Materials and methods:**

In a multicentric retrospective study, 375 SETK‑2 datasets of congenitally deaf children treated with CI from five centers were included. All children had been treated before the fourth year of life, and the interval between treatment of the two sides was less than 12 months.

**Results:**

In the analyses according to age and hearing age, all subtests in the observed groups, with the exception of word comprehension, showed significantly worse results than the normative values. Isolated results demonstrated results similar to or even better than the normative values. The more complex the tested performance, the higher the proportion of conspicuous test results. The timepoint of implantation had no significant influence on test performance.

**Conclusion:**

The SETK‑2 should be evaluated according to chronological age; otherwise, the progress of early speech development may be incorrectly assessed and interventions initiated too late. Moreover, evaluation according to hearing age disregards the child′s cognitive skills.

## Stand der CI-Versorgung

Für kongenital taube Kinder ist die Versorgung mit einem Cochleaimplantat (CI) die therapeutische Option, eine Hörwahrnehmung zu erlangen und somit den Lautspracherwerb zu ermöglichen. Es wird davon ausgegangen, dass sich die sprachliche Entwicklung der implantatversorgten Kinder im Verlauf der Entwicklung von Kindern ohne Hörschädigung angleicht [6, 31], sie also im Zeitverlauf ihren Entwicklungsrückstand aufholen. Ein wesentlicher Faktor scheint hierbei der Versorgungszeitpunkt zu sein. Je früher die bedeutungstragende Wahrnehmung von Schall möglich ist, desto besser sind die Prognosen bzgl. des Spracherwerbs [4, 15, 17, 21]. Erfolgt sie vor dem 18. Lebensmonat sind, deutlich bessere Leistungen bis hin zu altersgerechten Ergebnissen zu erwarten [8, 28, 32]. Die auch bei Kindern ohne Hörschädigung sichtbare Variabilität zeigt sich auch bei implantatversorgten Kindern [7]. Besondere Herausforderungen ergeben sich in den Bereichen Morphologie-Syntax, Textgrammatik [2] und bei den narrativen Fähigkeiten [29]. Da durch das CI keine Normhörigkeit erreicht werden kann, wird von einem im Vergleich erhöhten Risiko für Störungen der phonologischen und morphologischen Entwicklung ausgegangen [20]. Dies kann sich auch auf den Erwerb der Subjekt-Verb-Kongruenz auswirken [22]. Bei sehr langsamen sprachlichen Entwicklungsverläufen vermag eine Teilgruppe der implantatversorgten Kinder nicht aufzuschließen, es besteht das Risiko, im späteren Verlauf nicht ausreichend lautsprachlich erfolgreich kommunizieren zu können [7].

## Verlaufsdiagnostik bei CI-Rehabilitation

Eine gezielte Diagnostik und eine regelmäßige Überprüfung der Hör‑, Sprach- und Sprechfähigkeiten ist aufgrund der dargestellten Problematik durch eine verlaufsbegleitende Diagnostik indiziert [26].

Als allgemein anerkannter, standardisierter und normierter Test dient hier u. a. der „Sprachentwicklungstest für zweijährige Kinder“ (SETK-2) [10].

Die Problematik bei der Durchführung und Auswertung von Testverfahren mit hörgeschädigten und CI-versorgten Kindern besteht darin, dass keine allgemeingültige Einigkeit in Fachkreisen darüber besteht, ob die Auswertung von Testverfahren nach dem Lebens- oder dem Höralter (Zeitpunkt der beginnenden Hörerfahrung) erfolgen sollte. Wird das Testverfahren nach dem Höralter (HA) ausgewertet, hat das Kind einen kognitiven Entwicklungsvorteil gegenüber der Normstichprobe. Wird hingegen der Test nach dem Lebensalter (LA) auswertet, sind taube Kinder im Nachteil, da der Zugang zum Hören nicht seit Geburt bestand. Insofern wird derzeit meistens das HA als Referenzwert unter Einbeziehung des chronologischen Alters zur Testauswertung herangezogen [25].

Um dieses Dilemma anzugehen, wurden daher als erster Schritt die folgenden Fragen am Beispiel des Sprachentwicklungstests für zweijährige Kinder (SETK-2) in einer multizentrischen Studie untersucht: Wie entwickeln sich implantatversorgte Kinder im Vergleich zu Kindern ohne Hörschädigung hinsichtlich ihrer produktiven und rezeptiven Sprachverarbeitungskompetenzen? Ist als prognostischer Faktor das LA oder das HA entscheidend? Ist aufgrund der Untersuchung an einer großen Kohorte die Etablierung von Norm- oder Referenzwerten möglich?

## Studiendesign und Untersuchungsmethoden

Die retrospektive Datenerhebung erfolgte in einer multizentrischen Studie der Arbeitsgemeinschaft CI Rehabilitation e. V. (ACIR e. V.) im Zeitraum von 2001 bis 2021 [13]. Die gemeinsamen Daten 5 deutscher CI-Zentren wurden für einzelne standardisierte Testverfahren zusammengetragen, ausgewertet und in Einzelstudien bearbeitet. Die vorliegende Studie bezieht sich auf die Datenerhebungen des SETK‑2.

### Stichprobe

Eingeschlossen wurden kongenital taube Kinder, die bei CI-Versorgung nicht älter als 48 Monate waren. Bei sequenzieller Versorgung betrug der maximale Abstand der CI-Versorgung 12 Monate. Kinder mit Anomalien der Cochlea oder mit kognitiven Zusatzbeeinträchtigungen (soweit bekannt) wurden ausgeschlossen. Die regelhafte Insertion der Elektrodenträger war sichergestellt, alle Kinder sind an einem Zentrum des ACIR (Arbeitsgemeinschaft Cochlea-Implantat Rehabilitation) angeschlossen und nahmen an der entsprechenden Rehabilitationsmaßnahme teil. Eine ausreichende Kommunikationsfähigkeit (auf Deutsch), um die Sprachtestungen durchzuführen, und eine nachgewiesene auditive Diskriminationsfähigkeit zum Testzeitpunkt waren weitere Kriterien zum Studieneinschluss.

### Erhebungsinstrument und Testzeitpunkte

Der SETK‑2 ist ein normiertes und standardisiertes Testverfahren, das die Einschätzung der Sprachverarbeitungsfähigkeit in Bezug auf Verstehen und Sprechen zweijähriger Kinder ermöglicht. Das Testverfahren besteht aus 4 Untertests:

- Verstehen I: Wörter,
- Verstehen II: Sätze,
- Produktion I: Wörter,
- Produktion II: Sätze.

Eine prognostisch valide Beurteilung des Spracherwerbs wird durch Prüfung von sprachlicher Rezeption und Produktion auf Wort- und Satzebene erreicht. Hierdurch können Risikokinder identifiziert werden. Für die Auswertung und Beurteilung der Ergebnisse liegen Normwerte (T-Werte/Prozentränge) für die Altersgruppen (Gruppe I) von 24 bis 29 Monaten und von 30 bis 35 Monaten (Gruppe II) vor.

Der SETK‑2 schließt als Kriteriumsvalidität „echt bilinguale“ [10] Kinder mit ein, gemeint sind wohl simultan bilinguale Kinder [27], bezieht sich allerdings nicht auf früh sukzessiv bilinguale oder spät bilinguale Kinder oder Kinder, die 3 oder mehr Sprachen bzw. die Deutsche Gebärdensprache (DGS) als Erstsprache erwerben. Für eine korrekte Auswertung und Beurteilung müsste daher nicht der Wortschatz in einer Sprache, sondern der Gesamtwortschatz in allen erlernten Sprachen herangezogen werden [27]. Da dies praktisch mangels Sprachkenntnissen oft nicht möglich war und die zu untersuchende Kohorte möglichst homogen sein sollte, wurde eine Subgruppe monolingual deutschsprachiger Kinder separat ausgewertet.

Um weitgehend im Rahmen der Normierung (Durchführung im 3. Lebensjahr) zu bleiben, wurde regelhaft der Zeitpunkt der frühesten Testung gewählt, so denn mehrere Testungen vorlagen. Die Testzeitpunkte waren jeweils zentrumsspezifisch und daher nicht im Rahmen der Studie synchronisiert.

### Datenauswertung

Die gesammelten Daten wurden computergestützt mithilfe von Gnu R (General Public License der Free Software Foundation) ausgewertet, die Grafiken mit dem Paket ggplot2 [11] erstellt. Das Signifikanzniveau wurde mit 0,05 angenommen, wo sinnvoll, erfolgte die Testung mit einer Varianzanalyse („analysis of variance“, ANOVA) ggf. ergänzt um den Tukey-HSD-Test („honestly significant difference“) [23].

## Ergebnisse

### Stichprobenbeschreibung

Den Kriterien des Studieneinschlusses entsprachen 375 Kinder. Aus dieser Gesamtgruppe werden die Kinder identifiziert, die zum Testzeitpunkt zwischen 2;0 und –2;11 Jahre alt waren (Gruppe Lebensalter – „LA“) bzw. diejenigen, die zum Testzeitpunkt zwischen 2;0 und 2;11 Jahren Hörerfahrung aufgewiesen haben (Gruppe Höralter – „HA“). Die demografischen Daten der genannten Gruppen bitten wir Tab. 1 zu entnehmen.

**Tab. 1:** Stichprobenbeschreibung der Gesamtgruppe, unterteilt nach Lebensalter (LA) und Höralter (HA) in Jahren und Screening-Gruppe bzgl. Testkriterium

		Gesamtgruppe	Lebensalter	Höralter
Anzahl* n*		375	223	123
Geschlecht	m.	188	108	70
	w.	186	114	53
	k.E.	1	1	0
Ätiologie	Genetisch	117	–	–
	Infektiös	30	–	–
	Toxisch	3	–	–
	Unbekannt	213	–	–
	k.E.	12	–	–
Erstsprache	Deutsch	260	191	70
	DGS	14	4	5
	Andere	58	15	31
	k.E.	43	13	17
Implantationsalter	< 12 Monate	185	143	61
	12–24 Monate	147	73	44
	> 24 Monate	43	7	18
LA bei Erstaktivierung in Jahren	Min.	0,48	0,48	0,57
	Max.	4,10	2,37	3,33
	Median	1,01	0,93	1,01
	Mean	1,24	1,01	1,29
	1. Quartil	0,82	0,76	0,80
	3. Quartil	1,48	1,10	1,56
LA bei 1. Testung in Jahren	Min.	1,83	2,02	2,61
	Max.	7,71	2,99	6,15
	Median	2,87	2,61	3,49
	Mean	3,12	2,57	3,58
	1. Quartil	2,47	2,33	2,96
	3. Quartil	3,54	2,82	3,92
LA bei 1. Implantat in Jahren	Min.	0,39	0,41	0,47
	Max.	3,99	2,30	3,26
	Median	0,91	0,82	0,90
	Mean	1,13	0,90	1,19
	1. Quartil	0,72	0,67	0,72
	3. Quartil	1,35	0,99	1,46
LA bei 2. Implantat in Jahren	Min.	0,39	0,47	0,47
	Max.	4,51	2,84	4,15
	Median	1,09	0,93	1,17
	Mean	1,32	1,04	1,42
	1. Quartil	0,84	0,77	0,85
	3. Quartil	1,59	1,21	1,78
Durchschnittliche Differenz der Implantate in Jahren	Min.	0,00	0,00	0,00
	Max.	1,00	0,98	0,97
	Median	0,00	0,00	0,16
	Mean	0,19	0,14	0,23
	1. Quartil	0,00	0,00	0,00
	3. Quartil	0,305	0,24	0,39

*DGS* Deutsche Gebärdensprache, *k.E.* kein Eintrag, *m.* männlich, *Mean* Mittelwert,* Min.* Minimum, *Max.* Maximum, *w.* weiblich

Aus der Gesamtstichprobe wurde zusätzlich eine Subgruppe von hörgeschädigten Kindern gebildet, die monolingual deutschsprachig aufgewachsen sind (Subgruppe mono, Tab. 2). Hier wurde entsprechend wie beschrieben die Einteilung nach Höralter und Lebensalter vorgenommen.

**Tab. 2:** Stichprobenbeschreibung Subgruppe monolingual (mono) unterteilt nach Lebensalter (LA) und Höralter (HA)

		Subgruppe	Lebensalter	Höralter
Anzahl* n*		230	169	62
Geschlecht	m.	116	80	38
	w.	113	88	24
	k.E.	1	1	0
Ätiologie	Genetisch	64	–	–
	Infektiös	15	–	–
	Toxisch	3	–	–
	Unbekannt	142	–	–
	k.E.	6	–	–
Implantationsalter	< 12 Monate	131	112	39
	12–24 Monate	81	53	15
	> 24 Monate	18	4	8
LA bei Erstaktivierung in Jahren	Min.	0,48	0,48	0,57
	Max.	4,10	2,37	3,33
	Median	0,96	0,88	0,93
	Mean	1,13	1,00	1,14
	1. Quartil	0,77	0,75	0,75
	3. Quartil	1,33	1,10	1,23
LA bei 1. Testung in Jahren	Min.	1,83	2,02	2,61
	Max.	6,15	2,99	6,15
	Median	2,71	2,55	3,11
	Mean	2,84	2,56	3,38
	1. Quartil	2,38	2,33	2,90
	3. Quartil	2,99	2,80	3,75
LA bei 1. Implantat in Jahren	Min.	0,39	0,41	0,47
	Max.	3,99	2,30	3,26
	Median	0,85	0,78	0,83
	Mean	1,03	0,89	1,04
	1. Quartil	0,68	0,66	0,66
	3. Quartil	1,20	0,99	1,11
LA bei 2. Implantat in Jahren	Min.	0,39	0,47	0,47
	Max.	4,51	2,84	3,65
	Median	0,99	0,91	1,01
	Mean	1,17	1,01	1,21
	1. Quartil	0,77	0,75	0,75
	3. Quartil	1,38	1,12	1,34
Durchschnittliche Differenz der Implantate in Jahren	Min.	0,00	0,00	0,00
	Max.	1,00	0,98	0,90
	Median	0,00	0,00	0,11
	Mean	0,14	0,12	0,17
	1. Quartil	0,00	0,00	0,00
	3. Quartil	0,24	0,19	0,30

*k.E.* kein Eintrag, *m.* männlich, *Mean* Mittelwert,* Min.* Minimum, *Max.* Maximum, *w.* weiblich

### Ergebnisse des SETK-2

#### Gesamtgruppe

**Abb. 1:**
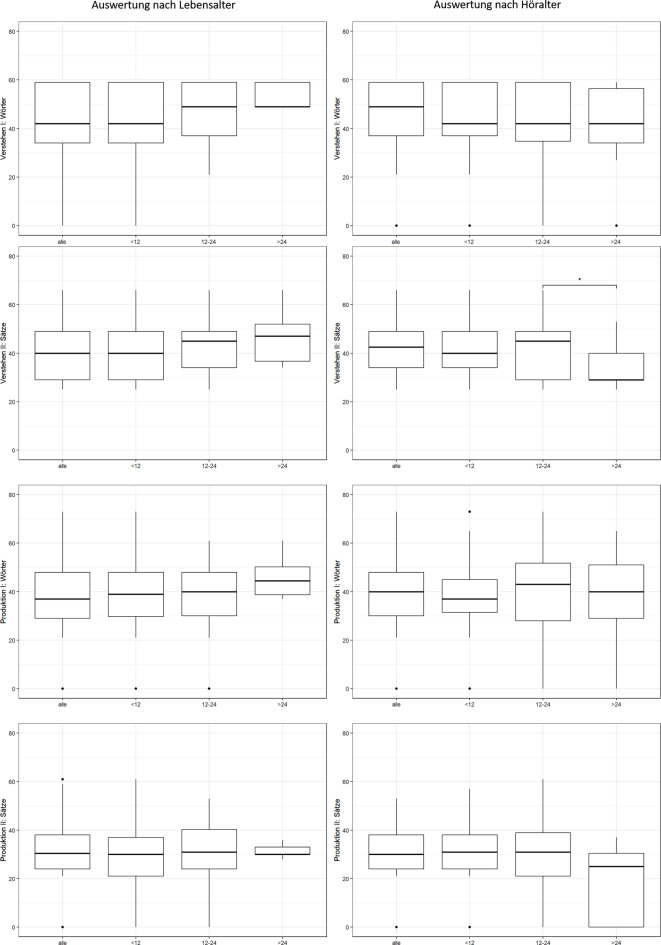
Gesamtstichprobe: Auswertung der Untertests des Sprachentwicklungstests für zweijährige Kinder (SETK-2): Verstehen I: Wörter, Verstehen II: Sätze, Produktion I: Wörter, Produktion II: Sätze, unterteilt nach Alter bei Implantation (in Monaten). Signifikante Unterschiede nur im Subtest „Produktion von Sätzen“, ausgewertet nach Höralter beim Implantationsalter 12–24 vs. > 24 Monate

Wertet man die Ergebnisse (Gesamtgruppe) des SETK‑2 im Rahmen der Gruppen nach LA (*n* = 223)/HA (*n* = 123) aus, ergibt sich das folgende Bild (Abb. 1): Je komplexer die Leistung, desto geringer der korrespondierende T‑Wert. Vergleicht man die Ergebnisse nach Implantationsalter – Aktivierung des Soundprozessors vor Vollendung des ersten Lebensjahres, im zweiten Lebensjahr oder später – zeigen sich bis auf den Untertest „Verstehen von Sätzen“ nach Höralter (Implantation im 1. oder 2. Lebensjahr, *p* = 0,047) keine signifikanten Unterschiede in beiden Gruppen (ANOVA, Tukey-HSD).

#### Monolingual deutschsprachige Kinder

**Abb. 2:**
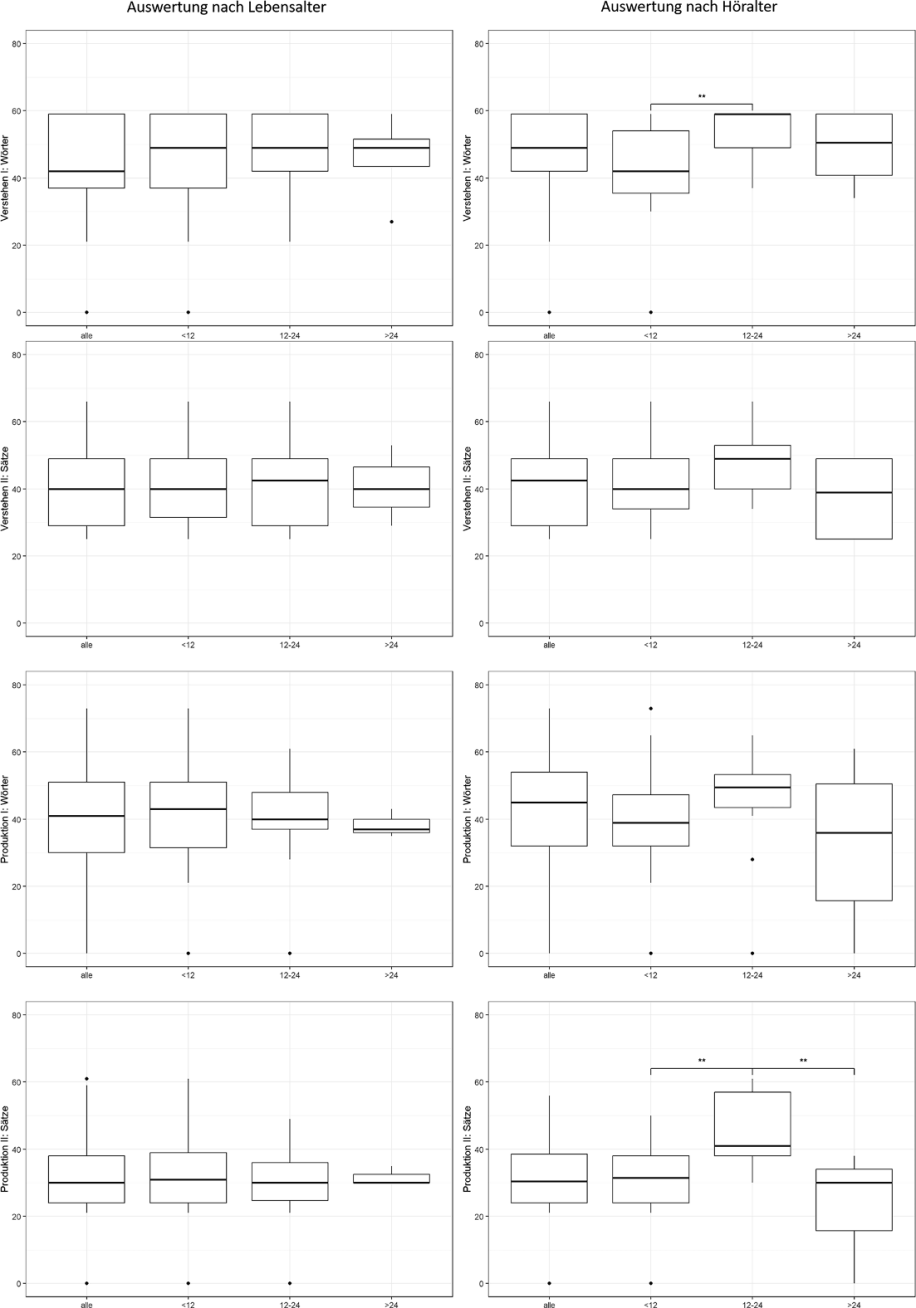
Subgruppe monolingual deutschsprachig: Auswertung der Untertests des Sprachentwicklungstests für zweijährige Kinder (SETK-2): Verstehen I: Wörter, Verstehen II: Sätze, Produktion I: Wörter, Produktion II: Sätze, jeweils gesamt und nach Implantationsalter

Wertet man die Ergebnisse der Subgruppe monolingual aus, zeigt sich im Wesentlichen das identische Bild. In den Auswertungen nach LA (*n* = 169)/HA (*n* = 62) schneidet die Gruppe HA, die zwischen 12 und 24 Monaten implantatversorgt wurde, beim Untertest Verstehen Wörter signifikant besser als die früh implantatversorgte Gruppe ab, bei der Produktion von Sätzen signifikant besser als die früh implantatversorgte und die später implantatversorgte Gruppe (ANOVA, Tukey-HSD; Abb. 2).

### Vergleich mit Normstichprobe

**Abb. 3:**
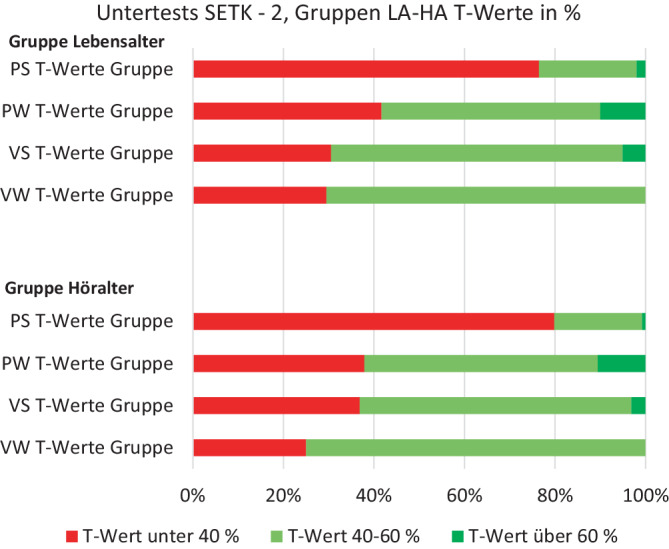
Subgruppe monolingual unterteilt in Gruppe Lebensalter (*LA*) und Höralter (*HA*). Anteil auffälliger T‑Werte in den einzelnen Subtests des Sprachentwicklungstests für zweijährige Kinder (SETK-2): *VW* Verstehen I: Wörter, *VS* Verstehen II Sätze, *PW* Produktion I: Wörter, *PS* Produktion II: Sätze, in der Auswertung nach LA und HA

Vergleicht man die einzelnen Subtests des SETK‑2 mit der zugrundeliegenden Normstichprobe der T‑Wert-Verteilung (MW = 50, SD = 10, t‑test), schneiden alle Gruppen bis auf den Untertest Verstehen von Wörtern, nach HA ausgewertet, schlechter als die Normstichprobe ab (Abb. 3 und Tab. 3 und 4).

**Tab. 3:** Auswertung der Gesamtstichprobe. T‑Werte der Untertests des Sprachentwicklungstests für zweijährige Kinder (SETK-2): *VW* Verstehen I: Wörter, *VS* Verstehen II Sätze, *PW* Produktion I: Wörter, *PS* Produktion II: Sätze, im Vergleich zur Normstichprobe in der Auswertung nach Lebensalter und Höralter

	Lebensalter			Höralter		
	MW	SD	*p*	MW	SD	*p*
VW	44,57	13,63	< 10^–8^	43,56	13,58	< 10^–7^
VS	41,18	10,82	< 10^–16^	40,09	10,60	< 10^–16^
PW	37,71	17,41	< 10^–16^	37,32	17,22	< 10^–14^
PS	27,26	16,35	< 10^–16^	26,08	16,48	< 10^–16^

*MW *Mittelwert, *SD* Standardabweichung

**Tab. 4:** Auswertung der Selektion von monolingualen Probanden. T‑Werte der Untertests des Sprachentwicklungstests für zweijährige Kinder (SETK-2): *VW* Verstehen I: Wörter, *VS* Verstehen II Sätze, *PW* Produktion I: Wörter, *PS* Produktion II: Sätze, im Vergleich zur Normstichprobe in der Auswertung nach Lebensalter und Höralter

Deutsch	Lebensalter			Höralter		
	MW	SD	*p*	MW	SD	*p*
VW	46,68	12,13	0,02	46,31	12,09	0,08
VS	41,26	10,62	< 10^–12^	42,71	10,73	< 10^–5^
PW	40,33	15,99	< 10^–9^	39,67	16,57	< 10^–5^
PS	28,66	15,24	< 10^–16^	29,59	16,36	< 10^–11^

*MW *Mittelwert, *SD* Standardabweichung

## Diskussion

### Rezeptive und expressive Leistung im SETK-2

Nutzt man den SETK‑2 zur Diagnostik des Sprachstands zweijähriger monolingual deutschsprachiger CI-versorgter Kinder, schneiden die Kinder in allen Subtests signifikant schlechter als die Normstichprobe ab. Die Auswertung nach Höralter vermag dies nicht auszugleichen. Während für die rezeptiven Aufgaben mehr als 60 % im Normbereich waren, verringert sich dieser Anteil für die Produktion von Wörtern, um schließlich im Subtest „Produktion von Sätzen“ – der komplexesten Leistung – einen deutlichen Spracherwerbsrückstand mit um 80 % auffälligen Leistungen zu zeigen.

In einer Studie von May-Mederake [18] wurden *n* = 28 mit CI versorgte Kinder mit einem Implantationsalter < 24 Monate mit dem SETK‑2 untersucht. Zwar erzielten alle CI-versorgten Kinder in allen Untertests im Mittel Leistungen im Normbereich. Jedoch erreichten die Kinder im Untertest „Produktion von Sätzen“ auch die schlechteste Performance, und nur eine geringe Anzahl der Gesamtstichprobe konnte in den Untertest miteingeschlossen werden (*n* = 6), sodass dies als nicht wirklich repräsentativ angesehen werden kann. Somit ist auch in dieser Studie eine auffälligere expressive als rezeptive Leistung mit einer Abnahme des Leistungsprofils von semantisch-lexikalischen hin zu morphologisch-syntaktischen Leistungen, wie auch in der vorliegenden Studie zu beobachten. Eine mögliche Erklärung für das schlechte Abschneiden im Untertest „Produktion von Sätzen“ könnte sein, dass Kinder mit CI im Vergleich zur Normstichprobe größere Schwierigkeiten haben, sich das Flexionssystem mit den wesentlichen Suffixen -t, -st, oder -n aufgrund ihrer eingeschränkten Hörwahrnehmung anzueignen und dadurch ggf. nur unzureichende Subjekt-Verb-Kongruenz erwerben [14]. Im Alltag haben sie aufgrund ihrer oft erschwerten Hörsituation Nachteile, weshalb Penke et al. [22] postulieren, dass hörgeschädigte Kinder für den Erwerb von wesentlichen Morphemen gezielten und qualitativ hochwertigen Input erhalten müssen, damit sie zu normalhörenden Kindern aufschließen können [25].

### Variabilität der Sprachentwicklungsverläufe

In vielfältigen Studien ist die Heterogenität der Sprachentwicklungsverläufe CI-versorgter Kinder dokumentiert [5, 8, 12, 24, 33]. Auch in der vorliegenden Studie (Abb. 3) beobachteten wir CI-versorgte Kinder, die zu normalhörenden Kindern aufschließen können, sog. Lückenschließer („gap closener“; [12]), oder sogar besser als die Normstichprobe waren. Diese Kinder können möglicherweise ihr präoperatives kognitives Erfahrungspotenzial mit der verbesserten Hörwahrnehmung für ein schnelleres Wortlernen nutzen [12, 16, 24, 25]. Mittlerweile wird eine schnellere Wortlernrate mit Testwerten „schneller als Höralter“ [25] als wesentlicher Hinweis für eine günstige Sprachentwicklung interpretiert [12].

Allerdings zeigen sich in der vorliegenden Studie auch Kinder, bei denen eine deutlich verlangsamte Sprachentwicklung auffällig ist. Inwieweit diese Gruppe die Meilensteine des frühen Spracherwerbs nicht bis deutlich verzögert erreicht, ist im Rahmen dieser Untersuchung nicht abschließend zu klären. Ein Verlauf lässt sich nicht darstellen, da der SETK‑2 nur für ein Lebensjahr normiert und eine Verlaufsdiagnostik über mehrere Jahre somit nicht darstellbar ist.

Zu den Faktoren, die zu einem altersgemäßen Spracherwerb führen können, zählen nach Boons et al. [1]: eine frühzeitige Erkennung und Versorgung der Hörschädigung und eine Teilnahme an einer CI-Rehabilitation (bei allen untersuchten Kindern gegeben). Allerdings sind ebenso der sozioökonomische Status der Eltern, Familiengröße und Bildungsstatus, die Qualität der Eltern-Kind-Interaktion und der elterliche sprachliche Input sowie die Elternbeteiligung an der Rehabilitation entscheidend. Dies wurde in der vorliegenden Studie nicht mitdokumentiert und könnte zum schlechteren Abschneiden beigetragen haben. Darüber hinaus wurden zum Zeitpunkt der Testung Zusatzbeeinträchtigungen ausgeschlossen, die sich eventuell erst zu einem späteren Entwicklungszeitpunkt hätten feststellen lassen, wie beispielsweise Sprachverarbeitungsproblematiken, die auch bei einem Teil der Normstichprobe vorliegen [12, 33]. Nach Reichmuth et al. [25] sollte bei früh versorgten Kindern ein verlangsamter Wortschatzerwerb – Beobachtungszeitraum 2 Jahre nach CI-Versorgung – kritisch betrachtet und entsprechend mit einer sprachfreien Überprüfung der nonverbalen kognitiven Fähigkeiten andere Ursachen ausgeschlossen werden.

Ein weiterer Faktor besteht möglicherweise in einem zu kurzen Beobachtungszeitraum: In einer Studie von Wie et al. [30] konnte bei 21 Kindern, alle im Alter zwischen 5 und 18 Monaten mit CI versorgt, im Rahmen einer Verlaufsdiagnostik erst 4 Jahre nach Implantation gezeigt werden, dass keine signifikanten Unterschiede bzgl. expressiver und rezeptiver Leistungen im Vergleich zur Normstichprobe bestanden. Auch May-Mederake u. Shehata-Dieler [19] postulieren einen Beobachtungszeitraum bis zum Lebensalter von 5 Jahren als guten Indikator für die Performance, da die Kinder mit CI bis zu diesem Zeitpunkt die Lücke zwischen Hör- und Lebensalter geschlossen hatten. Der kurze Normierungszeitraum des SETK‑2 erlaubt dies nicht, eine Kombination mit anderen Instrumenten wäre notwendig.

### Auswertung nach Lebens- oder Höralter

Das HA ist insgesamt als Bezugsmaß kritisch zu sehen, da die kognitiven präoperativen Erfahrungen nicht miteinbezogen werden. Der Aussagewert, dass ein z. B. vierjähriges Kind die Entwicklungsnorm eines zweijährigen Kindes erfüllt, erscheint aufgrund der auseinanderlaufenden kognitiven und sprachlichen Entwicklung fraglich. Weitere Probleme können die unzureichende Sensitivität und Spezifikation für Testverfahren, z. B. der Elternfragebogen ELFRA‑2, sowie die Vernachlässigung des tatsächlichen Entwicklungsstands des jeweiligen CI-versorgten Kindes sein [9].

### Bezugswert Implantationsalter

In einer Metaanalyse von Bruijnzeel et al. [3] konnte in einzelnen Studien ein Vorteil für ein Implantationsalter < 12 Monaten für linguistische Fähigkeiten nachgewiesen werden. Allerdings ließ sich das nicht auf die gesamte Sprachkompetenz übertragen. Andere Studien weisen auf eine Versorgung mit CI bis zum 18. Lebensmonat als entscheidendes Kriterium hin [28]. Genau für diese Gruppe, die zwischen 12 und 24 Monaten implantatversorgt wurde, finden sich sowohl in der Gesamtgruppe HA wie auch in der Subgruppe monolingual HA signifikante Effekte – allerdings nur in insgesamt 3 Untertests (Gesamtgruppe HA, Untertest Verstehen Wörter; Subgruppe monolingual HA, Untertests Verstehen Wörter, Produktion von Sätzen) – während bei allen anderen Untertests keine Effekte bzgl. Implantationsalter beobachtbar gewesen sind. Insofern lassen sich keine generellen Rückschlüsse bzgl. des Versorgungszeitpunkts ziehen. Ebenso ist ein Beobachtungszeitraum von einem Jahr, wie in der vorliegenden Studie, möglicherweise unzureichend, und signifikante Effekte zeigen sich erst zu einem späteren Zeitpunkt in der Verlaufsdiagnostik [19, 30].

## Ausblick

Weiterhin erreichen viele auch früh versorgte Kinder den altersgemäßen Sprachstand im 3. Lebensjahr nicht, sodass eine frühe Versorgung allein als nicht hinreichend für eine regelrechte Sprachentwicklung gesehen werden kann. Je nach Verlauf ist daher eine Intensivierung, in Ausnahmen (T-Wert > 60) aber ggf. auch eine Deeskalation der therapeutischen Förderung indiziert.

Die Nutzung des Höralters zur Beurteilung lässt die unabhängig vom Hören stattfindende kognitive Entwicklung außer Acht. Deshalb besteht die Gefahr, die Entwicklung substanziell zu unterschätzen und so eine frühzeitige Intervention zu vereiteln. Hierzu gehören auch Maßnahmen wie die Einführung von Deutscher Gebärdensprache (DGS), lautsprachbegleitender Gebärden (LBG) oder im Bedarfsfall unterstützte Kommunikation (UK).

In der therapeutischen Gemeinschaft wird häufig diskutiert, dass eine bei Retestung sichtbare Entwicklung wertvoll zur Darstellung von Fortschritten sein kann (insbesondere bei Auswertung nach Höralter). Inwiefern die Feststellung, dass der Spracherwerb eines vierjährigen Kindes dem eines zweijährigen entspricht, hier hilfreich ist, sei dahingestellt. Wir sind der Überzeugung, dass sich im therapeutischen Setting geeignetere Methoden finden lassen sollten, um Fortschritt darzustellen, die nicht das Erfüllen einer Norm suggerieren, die schlussendlich aber keine Anwendung finden kann.

Die qualifizierte Beurteilung des Spracherwerbs bleibt daher eines der wesentlichen Bereiche der Rehabilitation nach CI-Versorgung und erfordert eine langjährige Betreuung, um die Entwicklung individualisiert verfolgen und unterstützend begleiten zu können.

## Fazit für die Praxis

- Eine die Cochleaimplantat(CI)-Rehabilitation begleitende qualifizierte Diagnostik ist und bleibt wichtig, um eine Orientierung bzgl. des Entwicklungsstands der Kinder zu erhalten.
- Viele Kinder erreichen den altersgemäßen Sprachstand im 3. Lebensjahr nicht, sodass eine frühe Versorgung allein als nicht hinreichend für eine regelrechte Sprachentwicklung gesehen werden kann.
- Die Nutzung des Höralters zur Beurteilung lässt die unabhängig vom Hören stattfindende kognitive Entwicklung außer Acht. Die Auswertung sollte daher nach dem Lebensalter des Kindes erfolgen.
- Eine Auswertung nach Höralter birgt die Gefahr, die Entwicklung zu unterschätzen und frühzeitige Interventionen nicht zu veranlassen, wie die Einführung von Deutscher Gebärdensprache (DGS), lautsprachbegleitender Gebärden oder unterstützte Kommunikation.

## Data Availability

Die Rohdaten der Auswertung können aufgrund der einschlägigen Datenschutzgesetzgebung nicht zur Verfügung gestellt werden. Aggregierte Daten sind auf begründete Nachfrage an die Korrespondenzautorin nach Prüfung des Einzelfalls verfügbar.
